# Macrophage polarization: an important role in inflammatory diseases

**DOI:** 10.3389/fimmu.2024.1352946

**Published:** 2024-04-10

**Authors:** Min Luo, Fukun Zhao, Huan Cheng, Mu Su, Yuanmin Wang

**Affiliations:** The Third Affiliated Hospital of Zunyi Medical University, The First People’s Hospital of Zunyi, Zunyi, Guizhou, China

**Keywords:** macrophage, M1, M2, inflammatory disease, mechanism, therapeutic application

## Abstract

Macrophages are crucial cells in the human body’s innate immunity and are engaged in a variety of non-inflammatory reactions. Macrophages can develop into two kinds when stimulated by distinct internal environments: pro-inflammatory M1-like macrophages and anti-inflammatory M2-type macrophages. During inflammation, the two kinds of macrophages are activated alternatively, and maintaining a reasonably steady ratio is critical for maintaining homeostasis *in vivo*. M1 macrophages can induce inflammation, but M2 macrophages suppress it. The imbalance between the two kinds of macrophages will have a significant impact on the illness process. As a result, there are an increasing number of research being conducted on relieving or curing illnesses by altering the amount of macrophages. This review summarizes the role of macrophage polarization in various inflammatory diseases, including autoimmune diseases (RA, EAE, MS, AIH, IBD, CD), allergic diseases (allergic rhinitis, allergic dermatitis, allergic asthma), atherosclerosis, obesity and type 2 diabetes, metabolic homeostasis, and the compounds or drugs that have been discovered or applied to the treatment of these diseases by targeting macrophage polarization.

## 1 Introduction

**Figure 1:**
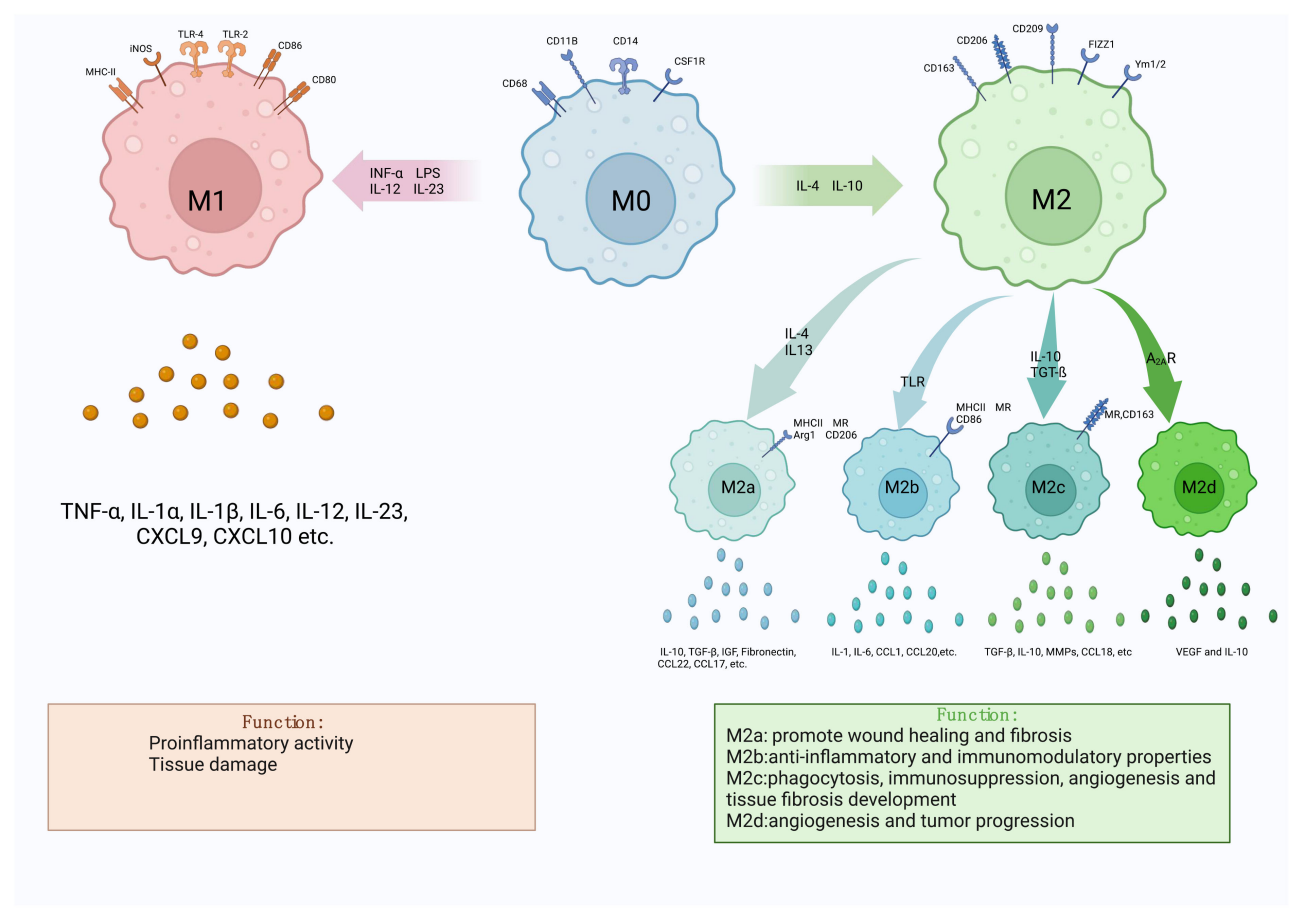
Diagram of macrophage polarization. There are two main forms of polarization of macrophages, M1-like and M2-like, which can be polarized into two types respectively under different cell stimulation conditions. M2-like macrophages can also be divided into four subtypes, M2a, M2b, M2c and M2d. Different macrophages have different markers on their surface, which can be used to identify the difference. The two types of macrophages secrete different cytokines during the disease process, resulting in two opposite effects.

Macrophages, as defined by Ilya Metchnikov in the nineteenth century, are phagocytic immune cells (1). It is a key component of innate immunity and plays a vital role in maintaining homeostasis and in recognizing and eliminating foreign pathogens. Research into the involvement of macrophages in the inflammatory response is burgeoning. Macrophages exhibit polarization and differentiation into distinct phenotypes in diverse inflammatory milieus, influenced by a plethora of factors. When exposed to lipopolysaccharide (LPS), interferon-α (INF-α), IL-12, IL-23, and other stimuli, resting macrophages (M0) were polarized into pro-inflammatory M1-like macrophages (M1). Conversely, IL-4 and IL-10 increased the development of anti-inflammatory M2 like macrophages (M2) (2, 3). According to various cytokines, M2 macrophages may be split into four different subtypes: M2a is generated by IL-4,IL-13 etc., M2b by immunoglobulin complexes and TLR agonists, M2c by IL-10,TGF-ß etc., and M2d is produced by M1 under the regulation of the activated adenosine 2A receptor (A_2A_R) (3). Among them, M2a, M2b and M2c were first proposed by Mantovani et al. according to different activation modes, while M2d was found to be a macrophage activated by toll-like receptors and specifically expressing vascular endothelial growth factor (VEGF) and IL-10 (4). M1 and M2 will coexist and be in balance under normal *in vivo* circumstances. When regional cytokines alter, M1 or M2 undergo reversible functional changes, allowing them to respond to inflammation *in vivo (* 5). Both types of macrophages have their own specific surface biomarkers, among which the main markers of M1 are CD80, CD86, TLR-4, etc., and the main markers of M2 are CD163, CD206, CD209, etc (6) (**Figure 1**). By secreting distinct cytokines, the four subtypes of M2-like macrophages also play distinct roles in different forms of inflammation and illnesses. The main receptors on the surface of M2a include MHCII, MR, Arg1, CD206, which can secrete IL-10, TGF-β, IGF, Fibronectin, CCL22, CCL17, etc., which can promote wound healing and fibrosis (7). MHCII, MR, and CD86 are examples of M2b surface receptors that can secrete IL-1, IL-6, CCL1, CCL20, and other substances. M2b exhibits potent anti-inflammatory and immunomodulatory properties. With MR And CD163 as surface markers, M2c can secrete TGF-β, IL-10, metalloproteinases (MMPs), CCL18, etc (8, 9). At the same time, it can play a role in phagocytosis, immunosuppression, angiogenesis and tissue fibrosis development. Finally, M2d is a macrophage that is activated by toll-like receptors and specifically expresses vascular endothelial growth factor (VEGF) and IL-10, also known as TAM, which is involved in angiogenesis and tumor progression (10–12). The table below summarizes a few M1 and M2 cell surface indicators as well as cytokines that encourage M0 polarization in both directions (**Table 1**).

**Table 1:** Factors that affect the polarization of macrophages.

Proteins/Gene	Normal Function	Effect on Polarization	Ref.
**Lipopolysaccharide**	Stimulants of the immune system	M1 polarization	(5, 6, 13–15)
**Interleukin-12 and Interleukin -23**	Cytokine	M1 polarization	(5, 6, 14, 15)
**Interferon-alpha**	Modulating immune response, antiviral	M1 polarization	(5, 6, 14, 15)
**Interleukin-4 and Interleukin-13**	IL-4 and IL-13 signaling	M2 polarization	(4, 5, 7, 8, 16)
**Toll-like receptors**	Non-specific immune response	M2 polarization	(4, 5, 7, 8, 16)
**Interleukin-10**	Cytokine	M2 polarization	(4, 5, 7, 8, 16)
**Transforming growth factor beta**	Cytokine	M2 polarization	(4, 5, 7, 8, 16)
**Adenosine A2a receptor**	G protein-coupled receptors	M2 polarization	(4, 5, 7, 8, 16)

By triggering the niacinamide adenine phosphate dinucleotide (NADPH) oxidase system and generating reactive oxygen species (ROS), M1-like macrophages aid in the removal of pathogens during infection. Consequently, ROS-induced tissue damage is mediated by M1-like macrophages, which also hinder wound healing and tissue regeneration. These macrophages have strong antibacterial and anticancer activity. On the other hand, mechanisms that restrict chronic inflammatory responses regulate the anti-inflammatory effects of M2 macrophages. M2 macrophages are involved in Th2 response, parasite removal, and inflammation inhibition. They also have a strong phagocytic capacity to remove debris and apoptotic cells, promote tissue repair and wound healing, angiogenesis, and fibrosis (16–18).

When the body is injured or exposed to an external bacterial infection, immune cells initiate the process of inflammation as a reaction. During this process, the body will mend itself by releasing numerous substances and recruiting immune cells. The duration of the inflammatory process varies from minutes to years (3, 19). An overly prolonged inflammatory response can lead to an imbalance between M1-like and M2-like macrophages, which can cause a number of problems, such as autoimmune diseases, diabetes, allergic diseases, atherosclerosis, obesity, metabolic homeostasis, and so on.

As a pivotal cell in innate immunity, macrophages play a significant role in various types of inflammation. This article primarily aims to offer an overview of inflammatory disorders closely linked to macrophage polarization and to delineate the current clinical approaches employed in treating these diseases.

## 2 Autoimmune disease

Autoimmune diseases are mainly caused by an immune response that misidentifies the host as an antigen (20). Over 80 autoimmune diseases have been identified, with nearly 5% of the world’s population affected (21). Due to the unknown pathogenic mechanisms of many diseases and the limited treatment options available, numerous drugs can lead to severe side effects, making autoimmune diseases a challenging condition for both doctors and patients (22, 23). Increased M1/M2 ratio is linked to a number of autoimmune illnesses, including rheumatoid arthritis, multiple sclerosis and experimental autoimmune encephalomyelitis, autoimmune hepatitis, inflammatory bowel disease and etc (24–26). The basic explanation for how macrophage polarization can contribute to some autoimmune disorders is that M1-like macrophages can create reactive oxygen species, which are essential to the autoimmune damage and repair processes (9).

### 2.1 Rheumatoid arthritis

Rheumatoid arthritis (RA) is a systemic chronic autoimmune illness that is the most prevalent autoimmune rheumatic disease, is more frequent in the elderly, and affects women more than males (27, 28). The primary feature of rheumatoid arthritis (RA) is its ability to induce synovitis in multiple joints, leading to joint swelling and other symptoms. However, prolonged illness can lead to cartilage destruction, joint necrosis, and ultimately disability (29). There are medications available to treat RA, such as nonsteroidal anti-inflammatory medicines, glucocorticoids, and disease-modifying anti-rheumatic drugs, however these medications must be taken for lengthy periods of time or at high dosages to be effective (30, 31). Nevertheless, this type of medication has also resulted in serious adverse reactions, indicating the need for new RA treatment strategies. Macrophages are important in the pathophysiology of RA. TNF-α, IL-6, and other inflammatory cytokines can be produced by M1-like macrophages, aggravating or maintaining inflammation, as a result, M1-like macrophages are also regarded as an important treatment target for RA (32, 33).

**Figure 2:**
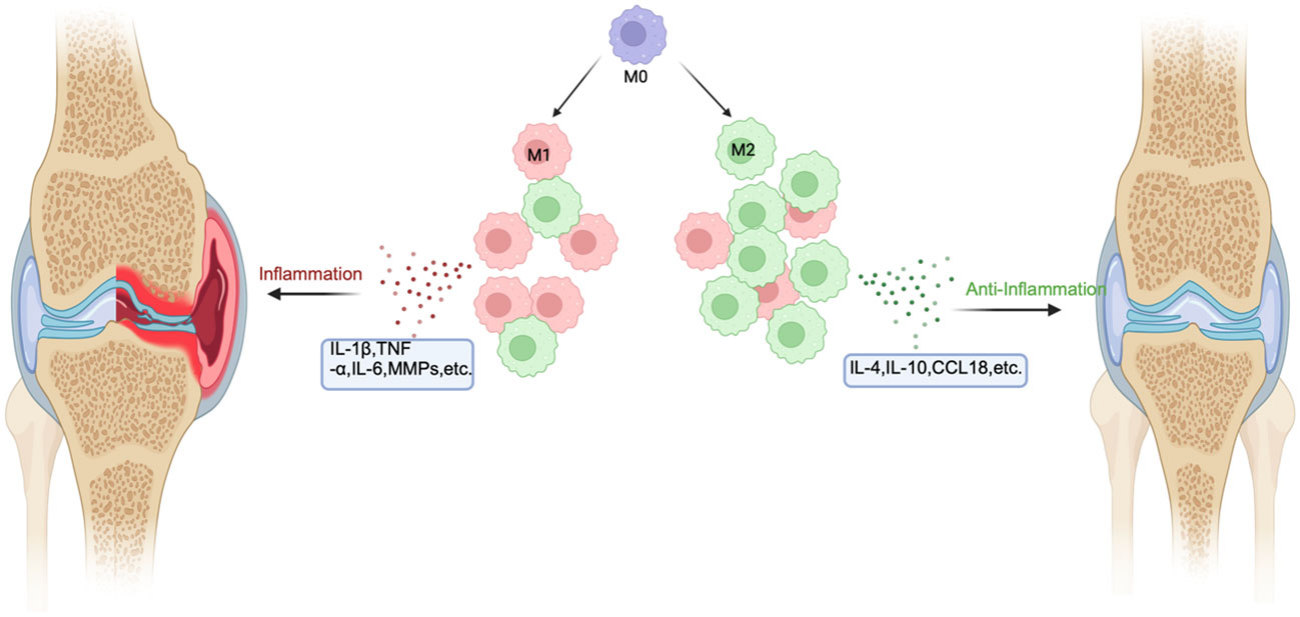
The mechanism of macrophage polarization in RA. In RA, the inflammatory environment will cause macrophages to polarize toward M1 and M2. In the area affected by RA, M1 is significantly more than M2. M1 can secrete inflammatory factors such as TNF-β and IL-6 to aggravate the disease, while M2 can secrete anti-inflammatory factors such as IL-10 to alleviate the disease.

High expression of M1-like macrophages is the main manifestation in rheumatoid arthritis, and macrophages with high expression can be detected in synovial tissue and peripheral blood of RA. M1-like macrophages in synovial tissue or peripheral blood primarily express major histocompatibility complex (MHC) class II, along with phenotypic markers such as CD80 and CD38, as well as inflammatory proteins like IL-6 and TNF-α. This enables them to present antigens to lymphocytes. Additionally, they can be recognized by T helper cells. Upon this recognition, they activate B lymphocytes to produce antibodies that are specific to the antigens presented by the macrophages, thereby thwarting pathogen invasion (32, 34). In RA, there is a response to invading pathogens, but persistent inflammation may lead to chronic maladaptive immune responses, leading to deterioration of the condition. The nuclear factor kappa B (NF-kB), stress-activated protein kinases/mitogen-activated protein kinases (SAPK/MAPK), and Janus kinase/signal transducer and activators of transcription (JAK/STAT) signaling pathways are connected to M1-like macrophage-induced inflammation in RA (35–37). These signaling pathways are primarily activated by pro-inflammatory cytokines, increasing macrophage survival (**Figure 2**).

An important indicator of RA remission is an increase in M2-like macrophages with anti-inflammatory properties, which indicates that the conversion of M1-like macrophages into M2-like macrophages is of the utmost significance for RA treatment. Although there is no drug on the market that specifically targets macrophages to treat RA, *in vitro* research has revealed that CTLA4-Ig (abatacept) can lower some cytokines made by M1-like macrophages, additionally, after receiving CTLA4-Ig, macrophages transform into anti-inflammatory M2-like macrophages (38). In addition, folic acid modified silver nanoparticles (FA-AgNPS) have been used for the treatment of RA specifically. By entering cells and releasing Ag, FA-AgNPS promote reactive oxygen species (ROS) clearance, M1-like macrophage apoptosis, and M2-like macrophage polarization (29). In other experiments, plasmids constructed with IL-10 pDNA and betamethasone sodium phosphate (BSP) were introduced into the biomimetic vector M2 exosome (M2 Exo), M2 Exo/pDNA/BSP significantly promoted the polarization of M1 to M2 (39). Targeted macrophage polarization therapy for RA remains to be studied, which is still a hot research direction in the future.

### 2.2 Multiple sclerosis and experimental autoimmune encephalomyelitis

One dangerous condition affecting the central nervous system is multiple sclerosis (MS), experimental autoimmune encephalomyelitis (EAE) is frequently utilized as an animal model of MS in pathological research because it shares many of the same clinical traits as MS (40, 41). It is an autoimmune-related neurodegenerative disease that can result in demyelination, inflammatory lesions of the CNS, and other symptoms. MS is a substantial contribution to non-traumatic disability in young individuals due to its chronic, localized, repeating, and diffuse nature (42, 43). The pathological phase of MS is associated with blood-brain barrier damage, T cell and macrophage infiltration into the CNS, activation of local microglia cells, and other variables. These acts, when combined, generate an inflammatory reaction that damages the neurological system (44, 45). Infiltrating macrophages and microglia have been identified as the primary effector of the inflammatory response in MS and EAE (46). Alterations in macrophages can influence a variety of inflammatory variables, resulting in alterations in the cellular environment, and the polarization balance of macrophages is critical in MS and other central nervous system illnesses (47).

**Figure 3:**
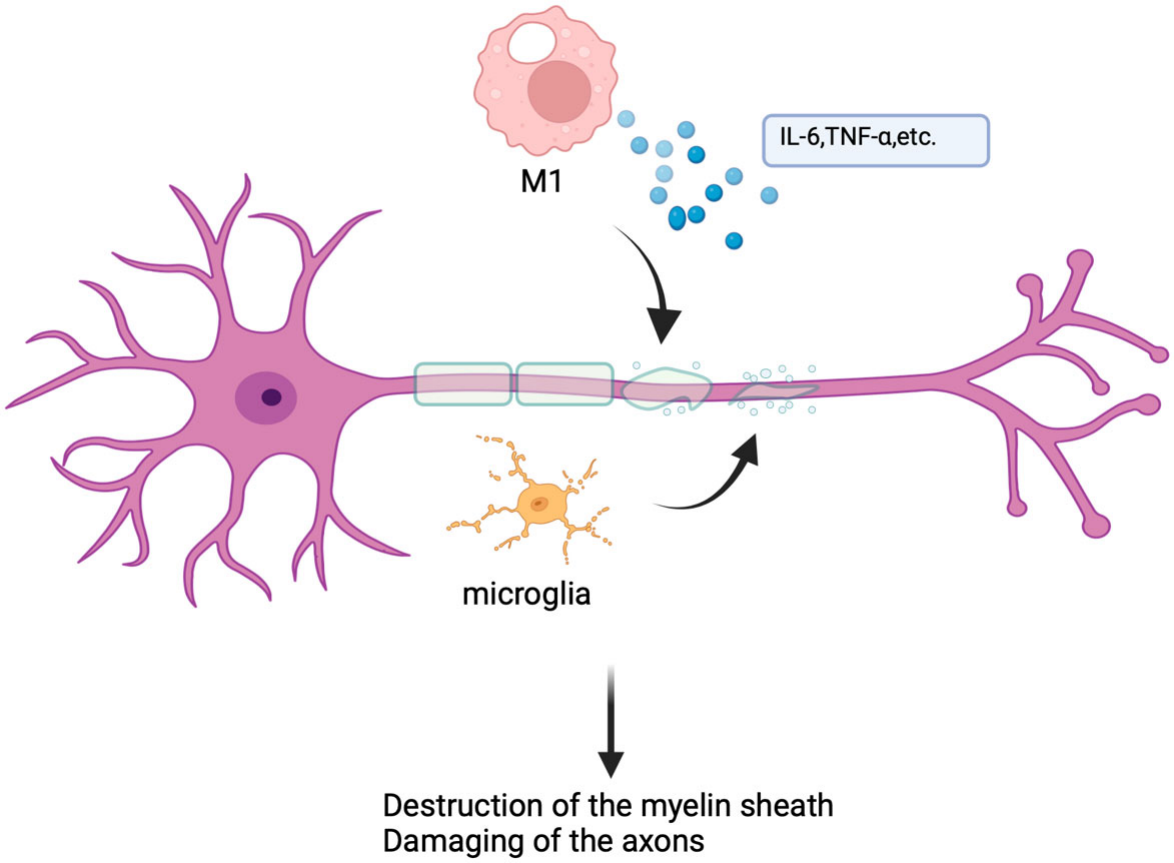
Mechanisms of macrophages in multiple sclerosis. In multiple sclerosis, microglia and macrophages play a major role in disease progression by secreting pro-inflammatory cytokines, presenting antigens, and inducing oxidative stress, leading to myelin destruction and axon damage.

*In vivo*, the development of MS and EAE is exactly complicated. M1 and M2like macrophages will arise in the body simultaneously. These cells must reduce inflammation, promote myelin repair, and phagocytosis of various debris while emitting inflammatory chemicals, causing oxidative stress, and damaging axons and myelin (48). During MS and EAE, there is a significant increase in iNOS, CD40, CD80, IL-6, and TNF-α. However, as the disease progresses, there is a shift toward M2-type macrophages taking over (47, 49) (**Figure 3**). It is known that taking myelin reduces inflammation and encourages the development of anti-inflammatory macrophages, demyelination is a significant inflammatory component of MS and EAE, and macrophages have a direct impact on it by removing myelin from the myelin sheath by phagocytosis (50, 51). Additionally, the blood-brain barrier’s (BBB) destruction is a significant contributor to neurological diseases, BBB can block the entry of harmful compounds into the CNS under normal physiological circumstances, whereas immune cells can pass through BBB (52). BBB is protected by microglial cells, macrophages, pericytes, etc., but when leukocyte extravasation causes inflammation, the perivascular macrophages of BBB will act as antigen-presenting cells to stimulate leukocyte recruitment to promote inflammation, this will also result in the recruitment of autoimmune T cells and accelerate the pathological process of MS through BBB (52–55). Furthermore, several additional investigations have discovered that certain proteins or non-coding RNAs can control the MS or EAE process by influencing the macrophage polarization process. For instance, studies have shown that the clinical and pathological severity of EAE is increased by the *in vivo* delivery of membrane protein Tim-3 antibody (56). Furthermore, circRNA_0000518 can exacerbate MS illness by promoting macrophage polarization toward M1 via the CaMKKβ/AMPK-PGC-1α pathway (45).

There is currently no effective treatment for MS. Maintaining physiological function following deterioration and lessening the degree of deterioration are the primary goals of therapeutic therapy for MS (57). Currently, it has been shown that several substances and proteins, such as diosgenin, glatiramer acetate, neuropeptide Y, valproic acid, etc., can treat MS and EAE by decreasing M1 or boosting M2 (49, 58, 59). Furthermore, as M2 macrophage regulators or to reduce inflammatory responses, 3,4-Disubstituted Piperidine Derivatives, M2 modulator B9, Baicalein, and grape seed extract (GSE) can be utilized to treat MS and EAE (60–63). Currently, a few polarizing therapies for MS are available on the market. These therapies primarily consist of two types of substances: proteins and peptides, such as interferon beta and ethyl acetate, and small molecules, such as glucocorticoids, dimethyl fumarate, and fingolimide/sibonimide (48). Many of these have been shown to indirectly modulate the polarization of macrophages and thus can be used as drug candidates to treat MS. Despite the fact that numerous medications have been discovered to treat MS and EAE, neither of them is yet fully curable. Thus, deeper study is required to find effective clinical treatments for MS and EAE in order to eradicate this terrible illness as soon as feasible. In addition, natural compounds are also a candidate that can be used to regulate the polarization of macrophages, and more experiments are needed to verify clinical drugs for the treatment of MS.

### 2.3 Autoimmune hepatitis

Autoimmune hepatitis (AIH) is a chronic inflammatory disease of the liver associated with the immune system, characterized by elevated levels of transaminases and immunoglobulin G (64). It was first defined as a chronic hepatitis in young women, but in recent years, the incidence of AIH has increased, the age distribution has widened, and AIH has also surfaced in the children (65, 66). Depending on the type of antibody it expresses, AIH can be divided into two classes: AIH-1 (positive for anti-nuclear antibodies or anti-smooth muscle antibodies) and AIH-2 (positive for liver-renal microsomal antibodies type 1, anti-LkM3 or anti-liver cytoplasmic antibodies type 1) (67). Clinically, AIH has no clear particular symptoms and may even exhibit subclinical features; nonetheless, if the initial start of AIH is not treated promptly, it is very simple to progress to liver failure or hepatocellular carcinoma (65, 68, 69). Despite being an extremely damaging autoimmune illness, the pathophysiology of AIH is still unknown due to its clinical variety (70). According to study findings, macrophages can initiate and control the inflammatory response of the liver, and persistent activation of macrophages can lead to liver disease, particularly in portal vein infiltration, where macrophage accumulation in AIH is strongly associated (71–73).

The histological characteristic of AIH known as interfacial hepatitis is caused by the Infiltration of monocytes, including macrophages. This highlights the significance of macrophages in AIH. The disruption of immunological homeostasis leading to Th0 cell development into Th1 cells significantly enhances macrophage activation. In AIH, more macrophages are activated, particularly in AIH-1, and these macrophages are primarily M1-like (74–76). It was found that macrophages showed aberrant activation in liver inflammatory disorders by identifying the macrophage activation marker soluble CD163, during acute AIH, the level of sCD163 was significantly increased, suggesting that macrophages play a role in the inflammatory process of AIH, and sCD163 may also serve as a marker for subsequent AIH targeting macrophage data (77). Although there has been research on macrophage activation in AIH, there are currently few publications on the relationship between macrophage polarization and the development and treatment of AIH. In the mouse experiment, zVAD was found to alter AIH through changing macrophage death, demonstrating the importance of macrophages in AIH (78). A potential therapeutic target for AIH is Receptor-interacting protein kinase 3 (RIP3), which can contribute to macrophage activation in AIH and promote IL-6 production, in addition, enhancer of zeste homolog 2 (EZH2) -mediated H3K27me3 may also influence the progression of AIH by modulating the polarization of macrophages, EZH2 is also promising as a therapeutic target (79, 80). Furthermore, studies have shown that polyguanine (PolyG) can bind to macrophage receptors with collagen structure(MARCO), and that polyG therapy can regulate MARCO, reduce M1-like macrophage polarization, and increase M2-like macrophage polarization, thereby linking AIH, MARCO can also become a target for AIH treatment (81). Interleukin is a frequent cytokine associated with a variety of diseases. IL-34, a member of the interleukin family, was reported to stimulate M2-like polarization of macrophages to suppress AIH in Concanavalin A-induced AIH (82).

The therapy of autoimmune hepatitis has not been investigated since the pathophysiology of the disease, particularly the unique involvement of macrophages in AIH, is not fully known. Since it has been demonstrated that IKK/NF-κB signaling activates chronic liver inflammation mediated by macrophages, medications that regulate the liver NF-κB system are a desirable therapeutic option to stop the onset of certain chronic liver disorders (72, 76). However, the importance of macrophages cannot be understated. They play a role not only in autoimmune hepatitis but also in other liver diseases. When a macrophage problem arises, such as in macrophage activation syndrome, it can cause liver enlargement, persistent fever, and damage to liver cells through the release of different cytokines (76). It’s clear that macrophages are involved in liver illnesses, and treating AIH using macrophages is a promising area for further investigation.

### 2.4 Inflammatory bowel disease

Inflammatory bowel disease (IBD), which consists of Crohn’s disease (CD) and ulcerative colitis (UC), is a major medical challenge throughout the world, particularly in Asia, where the number of cases has increased dramatically in recent years (83, 84). UC most frequently impacts the colon and rectum, whereas CD primarily affects the small intestine and terminal colon (85). Autophagy, genetic element, as well as environmental variables such as smoking, nutrition, medicines, and intestinal microbiota, all have an influence on the development of IBD (84). IBD is most frequent in teenage adolescents and early adults, where it is typically diagnosed, and is becoming more common in youngsters (86). The earliest clinical manifestations of IBD in children and adolescents mostly include stomach discomfort, diarrhea, rectal bleeding, perianal disease, and other symptoms that have a major influence on children’s growth (86, 87). Changes in macrophage homeostasis can affect IBD, such as sphingosine 1-phosphate receptor 2 (S1PR2) and its downstream G protein RhoA/Rho kinase 1 (ROCK1) signaling pathway can aggravate IBD by regulating polarization to M1-like macrophages, in addition, Yes-related protein (YAP) can inhibit M2-like macrophages and promote the production of IL-6 in M1-like macrophages to regulate the pathological process of IBD (88, 89). Instead, under the influence of diosgenin and TNF-α, respectively, miR-125a-5p and miR-24-3p can enhance the polarization of M2-like macrophages, relieving IBD (90, 91). In this review, we summarized the mechanism of macrophage polarization in CD and UC, as well as some clinical applications.

Crohn’s disease, a chronic inflammatory illness of the gastrointestinal system in humans, may cause lesions ranging from the mouth to the anus, as well as parenteral complications (92, 93). Since the 1960s, the pathophysiology of Crohn’s disease has been thought to be due to dysfunction of macrophages. Early in the inflammatory process, macrophages take absorb antigens, produce cytokines, and when their function is compromised, they are prominent in granulomas that serve as indicators for CD (94). Furthermore, in the presence of mucosal granuloma, M1-like macrophages are much more expressed in CD patients, suggesting that studying the innate immune system is more favorable to discovering the pathogenic mechanism of CD (95). According to research, the pathophysiology of CD is more likely to be associated to innate immune system abnormalities in the intestinal mucosa. The current findings imply that Crohn’s disease-associated Escherichia coli has been shown to block NF-κB signaling and thrive in macrophages (96). Infiltrating intestinal macrophages may generate significant levels of CD14 and other components, as well as activate NFκB and release molecules that promote inflammation, which leads to CD (97). In addition, 1,25 vitamin D has been demonstrated to serve as a CD14 ligand, inhibiting the generation of pro-inflammatory molecules (97). A protective function for protein tyrosine phosphatase non-receptor type 22 (PTPN22) in CD has also been shown by other investigations. Through its own inhibitory action, PTPN22 can increase the expression in M2-like macrophages and inhibit the expression in M1-like macrophages. It may also be controlled by AKT, and further research is still needed to determine its impact on macrophage polarization in CD (98).

Ulcerative colitis is a chronic illness of the intestinal mucosa that begins in the rectal area and progresses through the colon to the proximal end (99).The main clinical symptoms of UC are hematochezia, diarrhea and abdominal pain (100). UC affects both men and women equally and tends to be highly localized, occurring more commonly in Western nations. Many factors contribute to UC, including genetic factors and environmental variables, such as health problems from the environment, which promote changes in gut flora, which in turn contribute to UC (99, 101). The polarization imbalance of macrophages is a further significant factor in UC, and more and more research is being done on modulating macrophage polarization to relieve or cure UC.

In Crohn’s disease, the innate immune response exerts a greater impact compared to adaptive immunity due to the ability of macrophages to interact with T cells and initiate adaptive immune responses. The interaction between macrophages and T cells leads to the production of different cytokines that activate M1 and M2 macrophages (102). Additionally, individuals with Crohn’s disease often experience intestinal fibrosis, which is primarily caused by CD-associated adhering invasive Escherichia coli (AIEC), a pathogenic factor that targets macrophages as its main host cells (102, 103). AIEC exacerbates Crohn’s disease by specifically targeting macrophages. However, although M1-like macrophage polarization has been reported primarily in patients with CD (95), studies have shown that simple categorization of macrophages into M1 or M2 phenotypes does not fully explain their role in Crohn’s disease; these two types of macrophages are not mutually exclusive but rather coexist within the affected tissues. Reprogramming macrophages toward an M2 phenotype may not only fail to reduce the presence of M1 macrophages but could potentially worsen the disease progression (98, 102). Therefore, further research is needed to elucidate the mechanisms underlying the involvement of macrophages in Crohn’s disease and develop effective treatment strategies.

Several drugs have been found to improve Crohn’s disease in mice. For example, Loganin, a bioactive iridoid glycoside, was orally administered to mice and was found to alleviate various pathological abnormalities. Subsequent studies revealed that Loganin could significantly reduce the number of M1 macrophages in mice by regulating the sirt1/NFkB signaling pathway (104). Another example is Tiliroside, which promotes the proteasomal degradation of HIF-1α and down-regulates HIF-1α-dependent glycolytic enzyme mRNA expression in macrophages, thereby regulating M1/M2 macrophage polarization (105).Wu-Mei-Wan, Dioscin, and Shaoyao have all been reported to relieve ulcerative colitis by suppressing M1-like macrophage polarization by different study teams (106–109).

Macrophages play a significant role in the pathogenesis of many autoimmune disorders, and an unregulated or excessive immune response is thought to be a primary pathogenic component. The development of many autoimmune disorders is associated with the change of two types of macrophages. As a result, new therapeutic strategies targeting macrophage populations can help protect or mitigate a variety of autoimmune diseases; however, because the detailed mechanisms of treatment for some of these diseases are unknown, additional research is required to determine whether subsequent treatments targeting macrophages are a first choice for clinical treatment.

## 3 Atherosclerosis

Atherosclerosis is a chronic arterial wall disease that includes both innate and adaptive immune responses. It is the major cause of many cardiovascular disorders, including coronary artery disease, carotid artery disease, and peripheral artery disease. All phases of atherosclerosis are characterized by inflammation (110, 111). Inflammation has a role in the onset and progression of atherosclerosis. Endothelial damage, lipid metabolism abnormalities, and other issues contribute to the early stages of atherosclerosis (112). When endothelial cells are stimulated, inflammatory substances such as interleukin-8, intercellular adhesion molecule-1 (ICAM-1), and vascular adhesion molecule-1 (VCAM-1) are produced, which attract inflammatory macrophages and monocytes into the artery wall, causing inflammation (113). Monocytes that enter blood vessels differentiate into macrophages, which can transform into foam cells upon absorbing oxidized low-density lipoprotein (ox-LDL), potentially resulting in apoptosis. Subsequently, as foam cells accumulate near epithelial cells, they gradually develop into atherosclerotic plaques (114).Macrophages, as important players in inflammation and immune responses, also play an important role in atherosclerosis.

**Figure 4:**
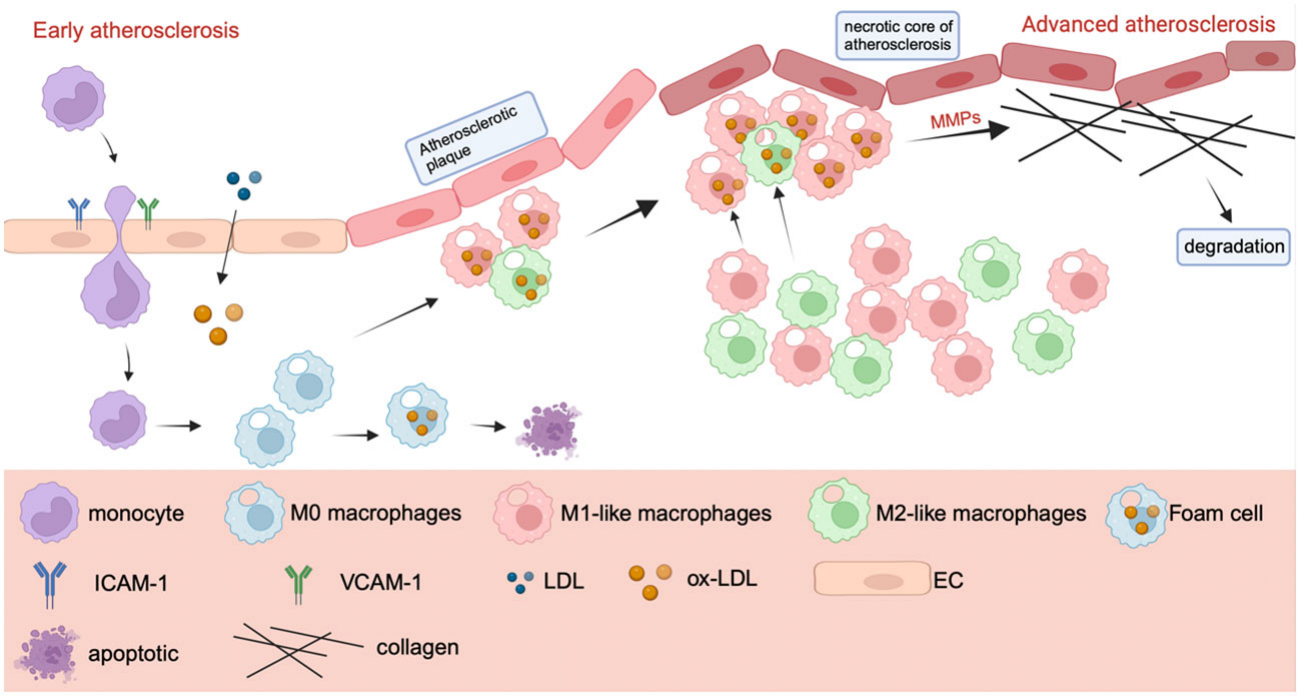
The mechanism of macrophage polarization in atherosclerosis. The mechanism of macrophage polarization in atherosclerosis. In the early stage of atherosclerosis, endothelial cells secrete ICAM-1 and VCAM-1 after stimulation to recruit monocytes and macrophages. Monocytes will gradually differentiate into M1 and M2-like macrophages. M1 and M2 macrophages transform into foam cells after uptake of ox-LDL, which can lead to cell death. In the advanced stage of atherosclerosis, macrophages secrete MMP, which leads to the destruction of collagen fibers, plaque rupture and bleeding, when collagen fibers are degraded, resulting in thrombosis.

The atherosclerotic necrotic core, initiated by the aggregation of foam cells, eventually forms due to subsequent inflammation. This inflammation releases more pro-inflammatory chemicals, continuously recruits additional monocytes, and directs macrophages to the lesion site (115–117). Atherosclerosis deteriorates because to the continual secretion of mechanism metalloproteinases by a significant number of macrophages in advanced atherosclerosis. MMPs destroy collagen fibers, causing plaque rupture, hemorrhage, and thrombosis (113, 118–120). **Figure 4** mainly shows a progression of atherosclerosis, in which M1 plays a major role in the deterioration process, while M2-like macrophages play a role in inhibiting plaque formation. The number of M2-like macrophages decreases gradually as the disease progresses (114, 121).

M1-like macrophages can be found in both early and late atherosclerosis. Many factors related to atherosclerosis, such as epigenetic modification, miRNA, autophagy and metabolic reprogramming, can also affect the polarization of macrophages (121). Plaques do not play a significant part in the condition, and the final cause of mortality is plate buildup, which leads to blood vessel obstruction. Macrophages are the most significant inflammatory cells in plaques, and the M1/M2 ratio regulates plaque growth and durability. Some investigations have shown that macrophage polarization can contribute to plaque development, providing a scientific foundation for atherosclerosis immunotherapy (122).

Because of the specialization of macrophages in the hardening of the central arteries, treating atherosclerosis by targeting macrophage polarization is a prudent strategy. Many chemicals or therapeutic treatments, including traditional Chinese medicine, have been discovered to be utilized or considered as prospective medications for the treatment of atherosclerosis by targeting macrophage polarization (123, 124). *In vivo* experiments in rats showed that Crocin can reduce oxidized low-density lipoprotein and inhibit atherosclerosis by inducing polarization of M2-like macrophages through vitamin D3 (125). Furthermore, ginsenosides Rb1 and Rg3 can reduce atherosclerosis by encouraging the polarization of M2-like macrophages (126). Other statins, such as sitagliptin and liraglutide for type 2 diabetes, or asperlin, have been demonstrated to reduce M1 macrophages following oral administration and are both useful in the treatment of atherosclerosis (121, 127, 128). The PI3K/Akt signaling pathway influences macrophage survival and proliferation, among other things. As a result, several research teams investigated the role of the signaling system in atherosclerosis and discovered that inhibitor therapy stimulates macrophage autophagy and greatly lowers atherosclerotic plaques in the early stage (129). Another study discovered that arsenic trioxide promotes macrophage autophagy through regulating ROS-dependent TFEB nuclear translocation and the AKT/mTOR pathway, lowering the risk of atherosclerosis (130). Other drugs that have been clinically proven to treat other diseases have also been found to regulate the phenotype of macrophages to treat atherosclerosis. For example, Kallistatin (a tissue kallikrein binding protein and serine protease inhibitor) can decrease M1-like macrophages (127, 131, 132). Many compounds that have an inhibitory effect on atherosclerosis are potential drugs for its treatment.

Based on preclinical models, plaque macrophages—the majority of plaque immune cells and the central cells of atherosclerosis—have positive effects by either decreasing the uptake of atherosclerotic plaques by macrophages, encouraging macrophage apoptosis, or polarizing macrophages more toward the M2 phenotype. It is desired to distribute therapeutic drugs selectively so they can directly modify the composition of macrophages. However, the degree and stage of the illness may determine how these therapies affect the disease’s course; if atherosclerosis is very severe, these treatments may have little or no effect.

## 4 Allergic disease

Allergic disorders may affect people of all ages, from babies to the elderly, and they frequently have a hereditary component. Rapid allergic reaction is the most prevalent form of allergic illness; its primary categories include cutaneous allergic reaction, respiratory allergic reaction, digestive allergic reaction, and anaphylactic shock. Among these allergic diseases, the most common ones include allergic rhinitis, allergic dermatitis, allergic asthma, and anaphylactic shock.

### 4.1 Allergic rhinitis

Allergic rhinitis (AR) is a highly prevalent condition that affects both adolescents and adults, and there is a hereditary component to the sickness. Allergic rhinitis, caused by the response of immunoglobulin E (IgE) to inhaled allergens, is one of the most common chronic diseases worldwide and is a very common chronic disease in high-income countries, with incidence of as high as 50% (133). It is also frequently associated with other conditions such as asthma, sinusitis, and conjunctivitis, all of which can impose a significant burden on patients, have a significant impact on learning, sleep, and quality of life, and have a significant economic impact on education, productivity, and the use of health care resources (134, 135).

A significant level of IL-4, IL-5, TNF-α, and other expressions are observed in allergies and seasonal rhinitis. These cytokines or inflammatory factors may originate from various cells, but they all contribute to the progression of inflammation (136). Macrophages may be implicated in the etiology of AR, and two non-coding RNAs have been discovered to influence macrophage polarization in allergic rhinitis by distinct study teams. The first research discovered that lncRNA-MIR222HG was considerably down-regulated in AR, and that it triggered the TRAF6/IKK/IB/P65 signaling pathway by targeting miR146a-5p, reducing M2-like macrophage polarization in AR and promoting AR development (137). Another study discovered that the lncRNA GAS5 blocked autophagy mediated by mTORC1/ULK1/ATG13 and activated the NF-kB signaling pathway, boosting M1 macrophage polarization and exacerbating allergic rhinitis (138).

### 4.2 Allergic dermatitis

Atopic dermatitis, a chronic inflammatory dermatitis characterized by eczema lesions and extreme itching, affects people of all ages and ethnicities and is a major contributor to the global burden of skin disorders (139). Atopic dermatitis affects roughly 20% of young children, and because it is difficult to entirely treat, it places a significant social and psychological burden on patients and families (139). Atopic dermatitis is also linked to an increased risk of a variety of comorbidities, including allergic rhinitis, food allergies, mental health issues, and asthma (140).

A high level of macrophage infiltration is linked to the development of atopic dermatitis, and M2-like macrophages also play an essential role in atopic dermatitis therapy (141). As a result, some research has discovered medications that can cure or alleviate atopic dermatitis by concentrating on the unique anti-inflammatory function of M2-like macrophages. *In vitro*, Hsa_circ_0004287 can enhance the ubiquitination degradation of S100A8/S100A9 in an M6A-dependent manner, therefore limiting the p38/MAPK pathway and, eventually, reducing the activation of M1-like macrophages, relieving skin inflammation in atopic dermatitis animals (142). Furthermore, many investigations have indicated that the flavonoids Naringenin and Diosmetin help reduce atopic dermatitis via influencing macrophage polarization. Naringenin can suppress M1-like macrophages and increase their polarization to M2, but Diosmetin can prevent macrophages from entering atopic dermatitis areas (143, 144). Another study found that Viola yedoensis Makino formula alleviates atopic dermatitis by activating JAK2/STAT3 signaling pathway and so on promoting M2 macrophages polarization (141).

### 4.3 Allergic asthma

Asthma is the most prevalent chronic respiratory illness, with allergic asthma being the most frequent kind, which is often defined by environmental allergens and it is characterized by bronchospasm and inflammatory infiltration (145). Allergic asthma has a far younger average age of start than other forms of asthma, accounting for over 90% of asthma cases in children and half of asthma cases in adults (146). Allergic asthma is defined by the presence of specific IgE sensitization to one or more aeroallergens, as well as proof of an allergen as the primary cause of asthma symptoms and management (147).

Macrophages constitute a significant fraction of immune cells in the lungs and play a vital role in asthma and other respiratory disorders induced by environmental causes. ATP6V0d2, a subunit of ATP6V0D2 that is abundantly expressed in macrophages, has been demonstrated to operate as an induced feedback inhibitor of asthma disease severity by boosting Pu.1 lysosomal degradation, which may contribute to the polarization of activated macrophages (148). When exposed to an allergen, the tissue adapts to its surroundings, polarizing the macrophages. Following an allergic reaction, M1-like macrophages produce cytokines and chemokines that help pathogen clearance and the recruitment of T and B cells. According to research, M1-like macrophages have greater expression levels in non-allergic asthma and are linked to severe asthma (149). On the contrary, M2-like macrophages, which are thought to be the primary macrophage in allergic asthma, have a stronger association (149). According to research, the LncRNA AK089514/miR-125b-5p/TRAF6 axis can enhance M2 macrophage polarization, which is important in allergic asthma. This also suggests a treatment target for allergic asthma (150).

Given the unique nature of M2 macrophages in allergic asthma, many studies have made efforts to identify substances that can be used to treat or alleviate allergic asthma by altering the polarization of M2 like macrophages, including glycosidic acid binding oligomeric domain like receptor protein 3 (NLRP3), autologous motion factor receptors (AMFR), and bone marrow-specific loss of fatty acid binding protein 5 (FABP5), which have been shown to promote the polarization of M2 like macrophages, thus achieving the effect of alleviating allergic asthma and providing treatment strategies for the prevention and treatment of allergic asthma (151–153).

Other allergic diseases, such as anaphylactic shock, occur very quickly and violently. Currently, there are few studies on the polarization of macrophages in these allergic diseases, which is also a direction of future research on allergic diseases.

## 5 Type 2 diabetes and obesity

Since the beginning of the twenty-first century, diabetes has been a severe worldwide public health issue. While the number of patients is increasing steadily in developed countries, it is accelerating virtually uncontrollably in developing nations (154). Type 1 diabetes (T1DM), also known as insulin-dependent diabetes, and type 2 diabetes (T2DM), also known as non-insulin-dependent diabetes, are the two primary kinds of the disease (155). T2DM is the most prevalent type of diabetes, accounting for around 90% of all patients, and it is predicted that 439.9 million people will have T2DM by 2030 (156). Nowadays, people’s lives have improved tremendously as a result of the fast growth of society and the economy. Eating habits heavy in salt, sugar, and fat have significantly raised the risk of diabetes, and people are more prone to suffer from T2DM owing to circadian rhythm disruption (157–159). Obesity, traditionally described as unhealthy weight caused by excess fat, is a disorder that is closely associated to diabetes. It has been confirmed that visceral obesity is closely related to insulin resistance, and insulin resistance caused by severe obesity can lead to metabolic disorders and even increase the risk of cardiovascular disease (160). M1 macrophages are known to be mostly glycolytic, whereas M2 macrophages are associated with TCA cycling and mitochondrial oxidative phosphorylation (161). As consequently, T2DM and obesity are metabolic disorders that unavoidably have a close association with macrophage polarization.

Type 2 diabetes is highly associated to inflammation as a chronic illness, and the occurrence and development of T2DM is closely tied to patients’ daily high-sugar diet, which also leads to various T2DM comorbidities such as periodontitis, obesity, and so on. T2DM increases the risk and severity of periodontitis substantially. Previous research has shown a high number of complement components in diabetic chronic problems. *In vivo* experiments in mice revealed that complement 3 (C3) is overactivated in T2DM-related periodontitis. C3 can participate in the immune inflammatory response, activating the up-regulation of IL-1, IL-6, and TNF-α, thereby activating M1 macrophages and inhibiting M2 macrophages. Periodontitis induced by T2DM can be avoided by lowering the concentration of C3 (162, 163). Other research has discovered that macrophages concentrate in the adipose tissue of obese patients, and obesity is linked to insulin-resistant T2DM. Weight increase causes local aggregation of inflammation and chemokines, as well as polarization of recruited monocytes into M1-like macrophages, enhancing adipose tissue dysfunction and impairing glucose tolerance, hence encouraging the incidence and progression of T2DM (164, 165). M1-like macrophages in T2DM and obese patients can produce lactic acid by promoting glycolysis rather than converting pyruvate to acetyl-CoA, whereas M2-like macrophages produce energy-rich molecules such as ATP via fatty acid oxidation and oxidative phosphorylation, and participate in the down-regulation of tissue repair and inflammation (166, 167).

Experiments in mice demonstrated that high-intensity interval training (HIIT) can reduce the polarization of M1-like macrophages, which is one of the primary mechanisms of M2-like macrophage polarization, and can therefore improve liver inflammation and lipid metabolism disorders in mice with T2DM. HIIT also demonstrated that mRNA expression levels of ROR and KLF4 were highly elevated in mice (168). Additionally, activation of the prostaglandin E2 receptor EP4 can shift the polarization of adipose tissue macrophages to an anti-inflammatory M2-like phenotype, limiting the inflammatory response of islets and maintaining beta cell function (169). Incidentally, in mice, Hyperoside was also found to inhibit T2DM-induced diabetic nephropathy by promoting the formation of M2 macrophages in mice (170).

**Figure 5:**
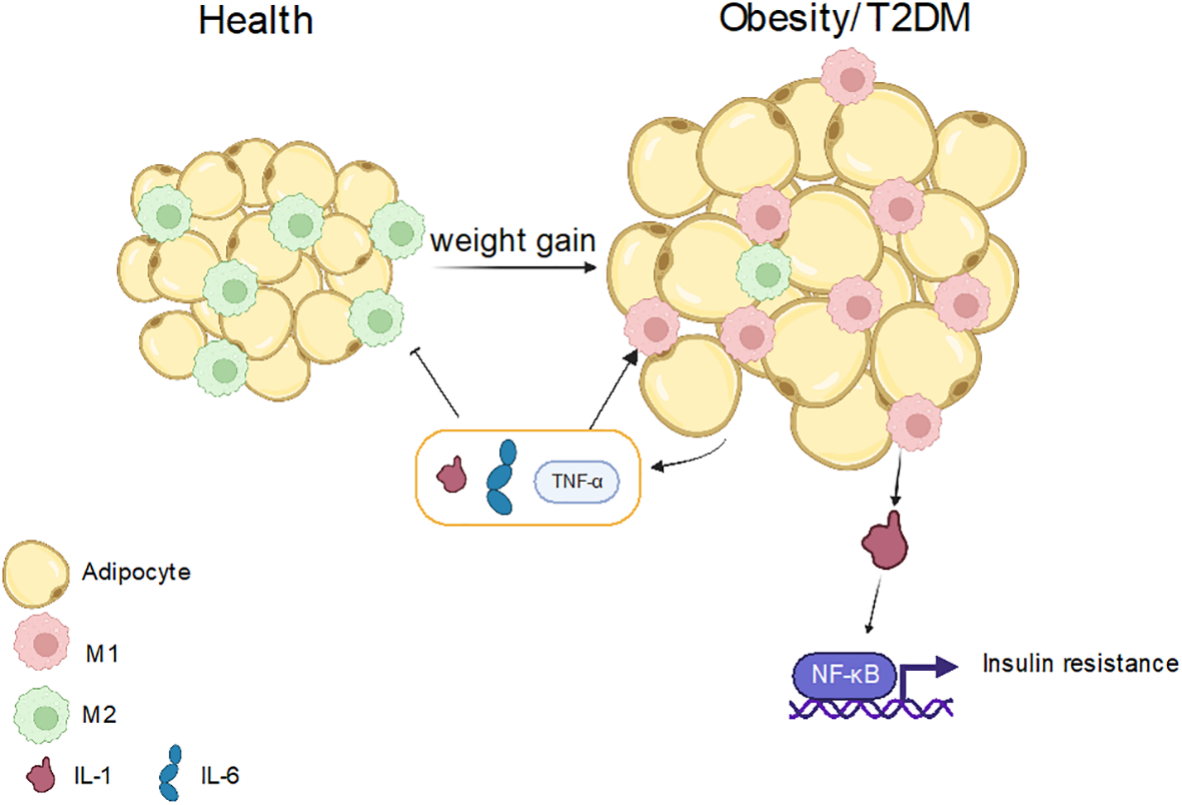
The role of macrophage polarization in obesity and T2DM. In normal people, fat cells are smaller, and the main macrophages are M2-like macrophages. When the body weight increases, the size of fat cells also increases, and M1-like macrophages are mainly in this case, and inflammation will secrete IL-1, IL-6 and other substances, which further promote the production of M1-like macrophages, but inhibit M2-like macrophages. It also produces IL-1β, which activates the NFkB pathway, leads to insulin resistance, and further promotes obesity or T2DM.

Obesity is increasingly prevalent in humans and is closely associated with the innate immune system. However, the mechanism of macrophage polarization in obesity differs significantly from that in type 2 diabetes mellitus (T2DM). Adipose tissue is susceptible to various inflammatory conditions in obese individuals as it serves as a major energy storage organ and a key site for inflammatory responses (171, 172). In obese patients, macrophages account for half of the innate immune cells in adipose tissue and are more likely to be polarized into M1-like macrophages. IL-1β, which is released by macrophages, can operate as an inflammatory cytokine, activating the NF-κB signaling pathway, phosphorylating the serine of insulin receptors, causing insulin resistance, and further promoting the progression of hyperglycemia in the body (171–174) (**Figure 5**). Moreover, studies have also found that miR-34a secreted by adipocytes can inhibit M2 polarization by inhibiting the expression of Kruppel-like factor 4 (Klf4), thus promoting obesity-induced inflammation (175). Recently, it has been found that autophagy can regulate immune response and macrophage polarization, while obesity can reduce the level of autophagy, make the polarization abnormal, and up-regulate the inflammatory response. It can be seen that obesity has a great impact on inflammatory diseases (175, 176).

Some studies have also found ways to alleviate obesity and T2DM by controlling or regulating the polarization of macrophages. For example, IL-25 can stimulate the polarization of M2-like macrophages, thereby promoting mitochondrial respiratory ability and lipolysis in adipose tissue to combat obesity (177). Sodium-glucose cotransporter (SGLT) 2 inhibitors were also found to improve obesity-related insulin resistance by regulating the balance of M1/M2 macrophages (178). In addition to this, activation of the SCAP-SREBP-1a pathway in macrophages may provide a novel therapeutic strategy to improve obesity by controlling cholesterol homeostasis in adipose tissue macrophages (179). At the same time, since obesity can also cause many complications, growth arrest-specific 6 (GAS6) can restore the phagocytic ability of macrophages and reduce the level of TUNEL and Caspase-3 positive cells, maintain cartilage thickness and prevent the progression of obesity-related arthritis. Therefore, targeting macrophage-associated ectocytosis or intraarticular injection of GAS6 is a potential therapeutic strategy for obesity-related OA (180).

Elevated glucose levels have a direct impact on macrophage polarization toward M1, and they can also stimulate the production of pro-inflammatory markers in macrophages. A high glucose concentration has a direct impact on macrophage polarization toward the M1 phenotype. With regard to the treatment and prevention of diabetes mellitus and its complications, the method of causing tissue macrophages to repolarize from a pro-inflammatory M1 phenotype to an anti-inflammatory healing M2 phenotype offers substantial potential.

## 6 Metabolic homeostasis

Metabolic homeostasis refers to the state in which the levels of intracellular metabolic substances, enzyme activity, and energy quality remain stable over time. Various factors such as environmental conditions, diet, physiological state, and other external influences play a crucial role in maintaining intracellular metabolic balance (181). *In vivo*, metabolic homeostasis is largely determined by the balance of metabolic pathways such as glycogen and glucose metabolism, and it is also linked to obesity and diabetes. The alternating activation and maintenance of relative balance of M1 and M2-like cells contributes to the maintenance of tissue homeostasis and host defense (182). Obesity is linked to metabolic homeostasis because numerous foods can directly engage in the signaling pathway of inflammation, and excess nutrition can readily trigger inflammation (182).

Numerous cytokines have been identified to impact cell metabolism and macrophage polarization, thereby playing a significant role in maintaining cellular homeostasis. Signal transducer and activator of transcription 6 are transcription factors that are activated by IL-4 receptor signaling. It can improve mitochondrial oxidative metabolism and hence macrophage activation by inducing the expression of peroxisome proliferator-activated receptor-coactivator 1 (183). Furthermore, Kruppel-like factor 4 can increase M2-like macrophage polarization, hence enhancing metabolic homeostasis (184). Beige fat in the human body may use energy while also resisting cold and obesity. IL-25 has been shown in studies to stimulate the production of Beige fat in cold environments, which has benefits for maintaining metabolic homeostasis, and IL-25 can cause the creation of Beige fat by alternatively activating M1 and M2 macrophages (185). It is clear that macrophage polarization is vital in metabolic balance. Other research has discovered that glucocorticoid receptor interaction protein (GRIP) 1, a major regulator of immune metabolism, can also govern macrophage polarization via several transcriptional pathways, promoting metabolic equilibrium (186).

In the human body’s metabolic organs, parenchymal and stromal cells play a role in preserving metabolic equilibrium. Macrophages are triggered in the body when there is an imbalance in nutrition due to bacterial and viral infections or symptoms like obesity. Obesity and type 2 diabetes are tightly linked to metabolic homeostasis. On the other hand, non-obese people have more M2 macrophages than M1 macrophages, and weight reduction is associated with the conversion of M1 to M2. Therefore, metabolic homeostasis can be regulated through the regulation of macrophages, but if you can lose weight with reasonable exercise at the beginning, you can reduce the inflammation of the body. If it is already very serious, weight loss coupled with drug treatment will also have a good effect.

## 7 Discussion and prospect

Macrophages are myeloid cells involved in innate immune responses, which originate from monocyte precursors in the blood and play a key role in tissue homeostasis under normal physiological conditions as well as after tissue injury (187). Macrophages are distributed in various tissues across the body and play a critical role in preserving internal balance and combating invading pathogens. Macrophages exhibit diverse morphological and functional characteristics as they undergo polarization in response to specific cytokines and growth factors present in the various tissues they inhabit (188). Macrophages are very heterogeneous and are composed of macrophages from a variety of different sources. So far, studies have found that macrophages can mainly differentiate into two phenotypes, pro-inflammatory M1-like and anti-inflammatory M2-like (5). During the inflammatory process, two types of macrophages undergo alternate transformations, and their proportions constantly change with the progression of the disease. This dynamic shift underscores the crucial role of macrophages in disease pathogenesis, making them an attractive target for therapeutic interventions. *In vivo*, macrophages have three main functions: phagocytosis, exogenous antigen presentation, and immune regulation. Both kinds of macrophages are stimulated alternatively during the inflammatory process. M1-like macrophages produce more and emit pro-inflammatory signals such as interleukin, restricting cell proliferation and causing tissue damage; subsequently, M2-like macrophages secrete more and play a mending function, aiding in inflammation reduction and wound healing. When the polarization of macrophages in the body is disrupted, it causes an imbalance in the M1/M2 ratio, which worsens inflammation in the body. A huge quantity of pro-inflammatory factors will set off a chain of events that will cause tissue and organ damage in the body (189, 190). As a result, if macrophage polarization can be properly regulated or changed, it will play an essential role in easing or curing a variety of disorders.

This review focuses on the role of macrophage polarization in various inflammatory diseases. The presence of abundant monocytes and macrophages in these diseases clearly underscores the pivotal role that macrophages play in inflammatory conditions. The various activation stages of macrophages are critical for the evolution of inflammation in atherosclerosis, metabolic balance, allergy illnesses, autoimmune disorders, type 2 diabetes, and other inflammatory diseases. In the early stages of many inflammatory disorders, M1-like is prominent, whereas in the later stages, M2-like is dominating. Several natural chemicals or molecules that can promote the polarization of macrophages to M2-like state have been found via ongoing research to regulate macrophage polarization. The polarization of macrophages can be harnessed to cure or alleviate numerous illnesses. Despite studies on macrophages in a variety of inflammatory diseases, research on their role in several allergy disorders remains limited. Many more studies are necessary for a complete understanding these diseases. Thus, future study on the impact of macrophage polarization on inflammatory disorders remains crucial.

Researchers are always looking for medications and chemicals that can modulate macrophage polarization in order to cure or relieve illness. Macrophages are an appropriate target for the remission or treatment of a variety of inflammatory illnesses. Some beneficial Chinese medicines, compounds, or other molecules have been discovered to have a regulatory effect on macrophage polarization. However, further research is required to develop specific drugs for clinical application. Moreover, further investigation is needed into the distinct mechanism of macrophage polarization in different inflammatory disorders. In various diseases, macrophages serve as a specific target, making it crucial and promising to explore the potential of treating other illnesses by modulating the polarization process of macrophages. In the last few decades, a wide range of disease models with inflammatory components—including cancer, joint sclerosis, kidney disease, and autoimmune—have been used to investigate therapeutic diagnostics related to macrophages. Future research will likely focus on using macrophages to cure diseases, so it is imperative that we learn more about the variations in macrophage formation, phenotype, and function. Researching the roles played by various macrophage subtypes in a range of illnesses and cancers can improve our understanding of how diseases arise and progress, influence treatment outcomes, and produce double the impact for half the work.

In recent years, significant progress has been made in the study of macrophages from the aspects of transcriptomics, transgenetics and epigenetics, among which the research results on macrophages in various inflammatory diseases are also of great significance for the clinical treatment of these diseases. However, due to the immature understanding of the mechanism of inflammatory diseases, the research on the treatment of macrophage polarization has been hindered. In addition, some people have proposed miRNA-based treatment methods, but these methods still need some basic research and other theories to verify. Despite the existing challenges in treating macrophage polarization, the extensive potential of this approach is evident from research findings, including validated methods in animal models. Subsequent studies should prioritize gaining a deeper insight into the disease pathology and the dynamics of macrophage polarization within the disease context. It is foreseeable that the clinical application of macrophage-based therapies for inflammatory diseases will undoubtedly become a reality in the future.

## Author contributions

ML: Writing – original draft, Writing – review & editing. FZ: Data curation, Formal Analysis, Writing – original draft. HC: Investigation, Writing – original draft. MS: Resources, Writing – original draft. YW: Funding acquisition, Supervision, Visualization, Writing – original draft, Writing – review & editing.
